# Design and Validity Evidence for a Unique Endoscopy Simulator Using a Commercial Video Game

**DOI:** 10.7759/cureus.18379

**Published:** 2021-09-29

**Authors:** Garrett Johnson, Ashley Vergis, Bertram Unger, Jason Park, Lawrence Gillman

**Affiliations:** 1 Surgery, University of Manitoba, Winnipeg, CAN; 2 Internal Medicine, University of Manitoba, Winnipeg, CAN

**Keywords:** surgical training, video games, simulator, endoscopy, education

## Abstract

Background

Procedural simulation enhances early endoscopy training. Multiple commercial simulators are available; however, their application is limited by cost and poor user compliance. First-person “shooter” (FPS) video games are popular. In this study, we aimed to show that a novel in-house designed colonoscope controller used to play an FPS video game shares similar constructs with real-life endoscopy.

Methodology

Participants completed the first three levels on an FPS video game, Portal (Valve Corporation, Bellevue, WA), first using a conventional controller and then the modified endoscope controller. A total of 12 expert endoscopists and 12 surgical residents with minimal endoscopy experience were evaluated based on completion time, button presses, and hand motion analyses.

Results

Experts outperformed novices for completion time (expert: 944 seconds; novice: 1,515 seconds; p = 0.006) and hand movements (expert: 1,263.1; novice: 2,052.6; p = 0.004) in using the novel colonoscope controller. There was no difference in button presses or total path length traveled. Furthermore, performance did not differ using conventional game controls.

Conclusions

Experts outperformed novices using the endoscope but not the conventional controller with respect to the economy of movement and completion time. This result confirms that our endoscope-controlled video game shares similar paradigms with real-life endoscopy and serves as a first step toward creating a more enjoyable and cheaper alternative to commercially available endoscopy simulators.

## Introduction

Surgical training is facing new challenges, including work hour restrictions [1-4], limited operative resources, increased concern for trainee supervision during procedures [5], and the explosion of new techniques and technologies used in daily practice [6]. These issues challenge our ability to train surgeons using traditional instructional models and timelines. Procedural simulation has been increasingly implemented as a core component of surgical training programs to combat these challenges, and courses employing simulations, such as Fundamentals of Laparoscopic Surgery (FLS) and the newer Fundamentals of Endoscopic Surgery (FES), have become popular [7,8]. For endoscopy, in particular, multiple commercial endoscopic simulators have been developed [9,10], and studies, including a recent Cochrane meta-analysis, suggest that these simulators can be used to supplement early endoscopy training [11,12]. However, these commercial simulators have several limitations, including high cost ($50-100,000 USD), as well as difficulties with compliance, particularly when access to the simulator is restricted to the workplace [13]. Thus, there is a need for low-cost, effective endoscopic training methods that learners are willing to practice at home in their free time.

Some authors have hypothesized that first-person “shooter” (FPS) video games can help develop early endoscopy skills [14]. The commercial video gaming industry continues to grow, with more adults becoming avid gamers. Overall, 61% of all Canadians play video games, with 50% being women. Furthermore, the average age of a video game player in Canada is 34 years [15]. Video games have been shown to increase visuospatial and attention skills [16,17] and may be associated with improved laparoscopic performance [18,19] and endoscopy skills [14,20,21]. Custom video games and controllers have been developed with the intent to supplement laparoscopic skills training [22]. However, to our knowledge, this approach has never been successfully employed for endoscopy.

We hypothesize that a challenge facing early endoscopy trainees is familiarization with the control head. By integrating a conventional endoscope as a controller for a commercially available video game and instituting a training regimen utilizing an FPS platform, we hope to create a cheaper training tool that will improve early endoscopy performance and that trainees will find fun to play. Therefore, the overall goal of this initial proof of concept study was to collect validity evidence for the use of a novel endoscopy training tool by demonstrating that trained endoscopists outperform novices using our controller.

## Materials and methods

Study design and theoretical framework

Messick’s validity framework was used as the underlying theoretical framework to guide this research. This framework has become the gold standard when evaluating validity evidence for performance assessments and is supported by multiple national and international education and research organizations [23]. In brief, the Messick framework consists of five sources of validity evidence: content, response process, internal structure, relations with other variables, and consequences of the assessment or test. We refer readers to a recent systematic review on this topic for further details regarding the use of this framework in surgical simulation [24]. According to this framework, we attempted to establish initial validity evidence by measuring participant performance in “relation to other variables.” We compared novices and experts on our new simulator and measured performance via time, button presses, and hand motion analysis (HMA) data.

Participants and sample

The novice group consisted of junior surgical residents with minimal or no personal experience with endoscopy. The expert group consisted of fully trained staff surgeons accredited by the Royal College of Physicians and Surgeons of Canada who are proficient in endoscopy and perform endoscopy in their usual practice. Participants were recruited by convenience sampling.

Previous skills training research suggests effect sizes ranging from 1.0 to 1.2 standard deviations between novices and experts [22,25,26]. A power calculation using a two-tailed test with a power of 0.8 and alpha of 0.05 estimated a need to recruit two cohorts of 12 participants each. In total, 12 experts (12 general surgeons) and 12 novice residents (nine general surgery, one urology, one plastic surgery, and one orthopedics) were included in the study.

Controller and video game

**Figure 1:**
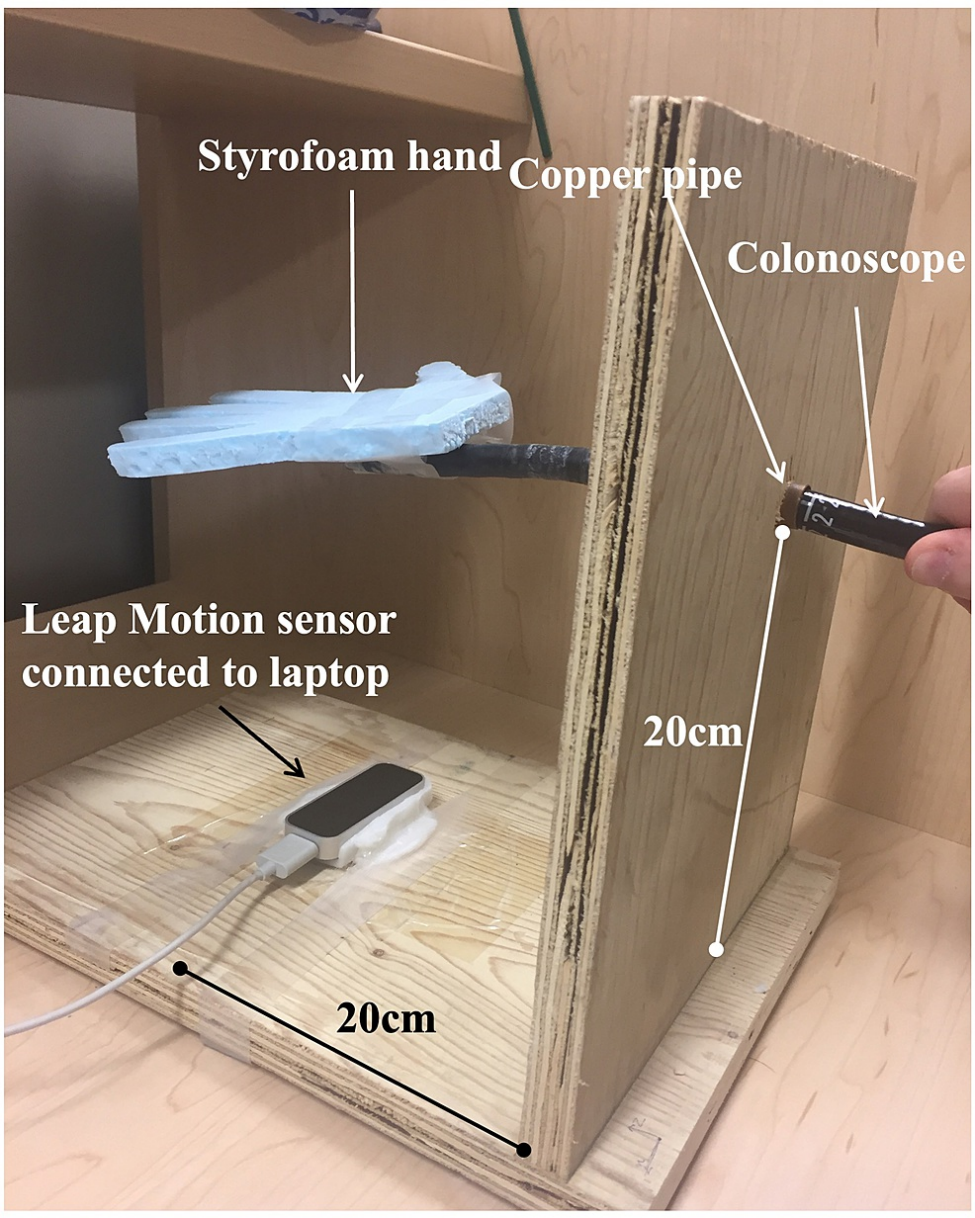
Modified colonoscope controller used as the video game controller to play first-person shooter video games.

A colonoscope without a camera, light source, or power was specially modified for this study and used as the controller to play the video game (Figure 1). A wooden platform was constructed with an opening in its center for a 10 cm copper pipe to simulate a natural orifice to house the device (Figure 1). The scope was advanced 18-20 cm through the copper pipe so that the tip of the scope was elevated 20 cm over a Leap Motion hand motion sensor (Leap Motion Inc., San Francisco, CA). Commercially available computer software (GameWave for Mac, version 1.5.5) was used to control computer functions using the position data from the Leap Motion controller. The Leap Motion controller is designed to optically detect hand position to allow its use in motion-controlled software applications. In our application, the colonoscope tip was sensed by the Leap controller, and position data from the controller was passed to the modified GameWave software which converted scope position to simulated mouse movements. Mouse movements were then used as inputs to control an FPS video game. To improve scope detection by the Leap Motion device and correct inconsistencies with tracking, a Styrofoam hand was affixed to the scope. Using this combination setup, the colonoscope controller could be used to play an FPS video game on a MacIntosh laptop computer (MacBook Air 13-inch, Mid 2012, using MacOS Sierra 10.12.6, Apple, Cupertino, CA). A standard USB keyboard was modified with all the keys removed except for the insert key and space bar and placed on the floor to create foot pedals to input the button presses required for the “shoot” and “grab” functions in the game. The software specifications used to modify the GameWave computer software to control the video game are available upon request.

The video game used in the study was Portal (Valve Corporation, Bellevue, WA), a primarily problem-solving game that employs FPS constructs. Participants were asked to play through the first three levels of the game as quickly as possible, first using the mouse and keyboard, and then using the novel colonoscope controller. The tasks that participants had to perform in the game include advancing forward, backward, and sideways in simulated three-dimensional environments; shooting large holes in walls (portals); lifting and placing blocks onto compressible buttons; and opening and progressing through doorways. This video game involves targeting walls and objects but does not involve any targeting of simulated people or characters. The custom gameplay settings in Portal’s software used during the study period are available upon request.

Study procedure

After informed consent was obtained, all participants were asked to perform an initial pre-study questionnaire exploring demographic information, including all past video game experience, and FPS video game experience, rated on a seven-point Likert scale to account for potential confounding related to video game familiarity. Participants were then shown a video (Video 1) orienting them to the game controls and levels of play, thus familiarizing them with the process and eliminating any impact of problem-solving difficulty on the results. To further control for problem-solving ability and past video game experience, participants were asked to play through the first three levels of the game as quickly as possible using a mouse and keyboard (i.e., conventional controller) before using the modified colonoscope controller. A single researcher (GJ) was on hand throughout all trials to monitor and troubleshoot any technical difficulties with the computer software. At the completion of the study, participants were asked which controller they preferred. Video 2 shows one of the study investigators using the controller to play through the game.

**Video 1:** Instructional video provided to study participants for orientation to colonoscope and game controls.

**Video 2:** Colonoscope controller being used to play a video game.

The primary outcomes compared were time to task completion, total distance traveled, and the number of discrete movements, as assessed by HMA. These are previously validated metrics for expertise in procedural tasks, including endoscopy [25,27-30]. The number of keystrokes was also assessed as we hypothesized that less experienced operators may press buttons unnecessarily.

Hand motion analysis

Electromagnetic sensors (8 mm) (Ascension Technology Corporation; Burlington, VT, USA) were applied to the dorsum of participants’ hands at the mid-shaft point of the third metacarpal. Data on each sensor’s position and orientation (6-degree freedom, 0.5 mm position, and 0.1-degree orientation resolution) were collected using commercially available software (Cubes; Ascension Technology Corporation; 240 Hz sampling frequency). Data were interpreted using Motion Analysis and Recording Software version 3.0 (MARS3) developed by our institution (University of Manitoba Clinical Learning and Simulation Facility; Winnipeg, Manitoba, Canada). Clinically insignificant background movements from hand tremors were eliminated via a Gaussian filter set to a frequency cut-off of 1.666 Hz. Registration of a discreet movement is triggered by a change in velocity greater than 15 mm/s. These variables are based on previously validated thresholds [25,31]. As a quality control measure, calibration was confirmed prior to each study session by analyzing a predetermined pattern of movements and assessing for 100% agreement between the output from HMA software and the manual counting of an investigator’s movements. For assessment of HMA using the mouse and keyboard controls, we were unable to reliably calibrate the left-hand sensor (controlling the keyboard), as electronics from the study laptop interfered with the electromagnetic field measured by the HMA instruments; hence, only the right hand (controlling the mouse) was measured for the conventional control trials.

Statistical analysis

Statistical comparisons were performed using SPSS Statistics software version 27 (IBM Corp., Armonk, NY, USA). The mean time, the number of button presses, hand movements, and hand path length data were compared between each group utilizing the Mann-Whitney U test as data were not normally distributed. Normality was tested using the Shapiro-Wilk test and visually with a Q-Q plot and histogram. Homogeneity of variance was determined by Levene’s test. Statistical significance was set at α = 0.05. To assess the effects of self-reported video game and FPS experience, these data were compared to HMA outcomes via the Spearman correlation coefficient. Past colonoscopy experience was compared to HMA outcomes via a Pearson correlation.

## Results

Participant characteristics including past endoscopy, endoscopy simulator, video game, and FPS game experience are described in Table 1. Novices were significantly younger and had more current video game experience than experts; however, populations did not differ in total video game use, game use at any other age, or in FPS experience. All participants, including the left-handed operators, held the scope the same way, with their left hand controlling the dials and their right hand directing the tube.

**Table 1:** Participant demographics FPS: first-person shooter P-values < 0.05 are statistically significant. Values represent means except where indicated by *. Values for experience, endoscopies performed, and simulator uses are participation estimates. ^1^ Seven-point Likert scale (1 = no use, 7 = daily use); ^2^ minimum score 4 (never played), maximum score 28 (daily play throughout all ages); ^3^ n = 10, two experts declined to answer this question. ^W^ Independent sample Welch’s t-test; ^F^ Fisher’s exact test; ^U^ Mann–Whitney U-test (all two-tailed).

Comparator	Novice cohort n = 12, (range)	Expert cohort n = 12, (range)	P-value
Age	27 (24–30)	38 (32–45)	<0.001^W^
Males	6*	11*	0.069^F^
Right-handed	11*	11*	1.000^F^
Video game experience^1 ^currently	3.2 (1–6)	1.7 (1–5)	0.022^U^
Video game experience^1 ^ages 1–6	1.9 (1–5)	1.3 (1–3)	0.252^U^
Video game experience^1 ^ages 7–12	4.5 (1–7)	3.7 (1-7)	0.360^U^
Video game experience^1 ^ages 13–18	3.9 (1–7)	3.8 (1–7)	0.780^U^
Total video game experience^2^	3.3 (1–7)	2.6 (1–7)	0.155^U^
FPS experience^1 ^currently	2.2 (1–5)	1.3 (1–2)	0.122^U^
FPS experience^1 ^ages 1–6	1.2 (1–3)	1 (1)	1.000^U^
FPS experience^1 ^ages 7–12	2.4 (1–7)	2 (1–6)	0.358^U^
FPS experience^1 ^ages 13–18	3.3 (1–7)	2.8 (1–5)	0.639^U^
Total FPS experience^2^	2.3 (1–7)	1.8 (1–6)	0.264^U^
Total lifetime endoscopies performed	1.8 (0–10)	930 (200–2,800)^3^	0.007^W^
Total lifetime endoscopy simulator uses	0.2 (0–1)	4.8 (0–10)	0.005^W^

Using the modified colonoscope controller, experts completed the three video game levels with fewer total movements (expert: 1,263.1; novice: 2,052.6, p = 0.004) and in less time (expert: 944 seconds; novice: 1,515 seconds; p = 0.006) compared to novices. The number of left-hand but not right-hand movements was also smaller for experts versus novices. Experts and novices did not differ for the number of button presses or total path length traveled (Table 2).

**Table 2:** HMA outcome measures using colonoscope controller. SD: standard deviation; HMA: hand motion analysis P-values < 0.05 are statistically significant. ^U^ Mann–Whitney U-test, two-tailed.

	Left hand, Mean (SD)			Right hand, Mean (SD)			Combined score, Mean (SD)		
Outcome measure	Novice	Expert	P-value	Novice	Expert	P-value	Novice	Expert	P-value
Time, seconds	-	-	-	-	-	-	1,515 (542)	944 (300)	0.006^U^
No. of movements	1,173.0 (467.8)	644.8 (230.0)	0.002	879.6 (383.4)	618.3 (247.0)	0.089	2,052.6 (815.5)	1,263.1 (457.5)	0.004^U^
Path length traveled, m	666.3 (406.2)	393.1 (252.4)	0.089	561.7 (407.4)	364.2 (296.2)	0.160	1,228.0 (702.2)	757.3 (456.2)	0.143^U^
No. of button presses	-	-	-	-	-	-	66 (23.5)	60 (16.4)	0.580^U^

There were no differences between novices and experts using the conventional mouse and keyboard controls (Table 3).

**Table 3:** HMA outcome measures using conventional (mouse and keyboard) controller. SD: standard deviation; HMA: hand motion analysis P-values < 0.05 are statistically significant. ^U^ Mann–Whitney U-test, two-tailed.

Keyboard score, Mean (SD)			
Outcome measure	Novice	Expert	P^U^
Time, seconds	185.7 (44.1)	199.3 (41.1)	0.298
No. of movements	40.7 (28.4)	28.8 (12.4)	0.385
Path length traveled, m	19.22 (10.26)	12.33 (6.03)	0.052
No. of button presses	28.9 (13.1)	31.8 (23.5)	0.450

**Figure 2:**
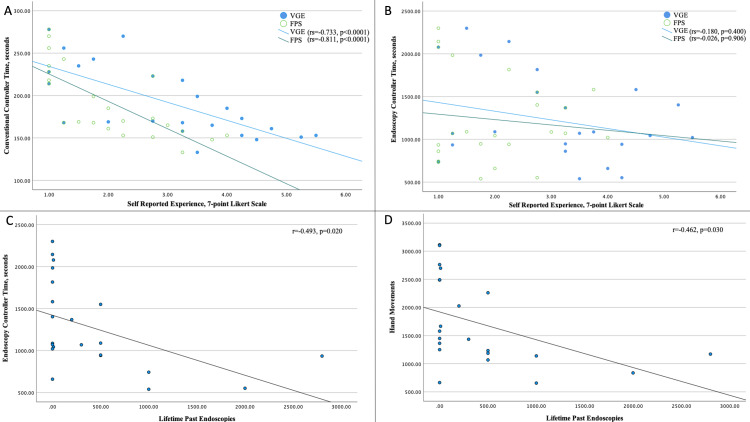
Scatter plots showing (A) self-reported VGE and FPS experience versus time using mouse and keyboard. (B) VGE and FPS versus time using colonoscope controller. (C) Self-reported past endoscopy experience versus time using colonoscope controller. (D) Past endoscopy experience versus total hand movements using the colonoscope controller. VGE: video game experience; FPS: first-person shooter

Self-reported total lifetime video game experience and FPS game experience were both significantly correlated with time to completion using standard mouse and keyboard (rs = -0.733, p < 0.0001; rs = -0.811, p < 0.0001, respectively; Figure 2A) but were not associated with time to completion using the colonoscope controller (rs = -0.180, p = 0.400; rs = -0.026, p = 0.906, respectively; Figure 2B). By contrast, self-reported number of past endoscopies was moderately correlated with time to completion (r = -0.493, p = 0.020) and total hand movements (r = -0.462, p = 0.030) (Figures 2C, 2D) but not path length (r = 0.350, p = 0.110; not shown) using the colonoscope controller.

## Discussion

Simulation will continue to play an important role in resident training, given work hour restrictions and patient safety concerns. Existing simulators have multiple limitations, including lack of portability, high cost, and poor compliance [13]. This novel video game controller could provide trainees opportunities to practice at home in their spare time while engaging in a popular recreational activity that many might otherwise enjoy. Traditionally, endoscopy training has relied upon meeting arbitrary volume thresholds as surrogate markers for competence to attain licensure. However, as modern training paradigms transition their focus to meeting competency thresholds, opportunities to practice skills outside of the confines of work-hour restrictions will be an asset [32].

This study was designed to provide validity evidence by demonstrating that performance on the device can differentiate between novices and experts using multiple measures. The endoscope handle-based controller can differentiate between novices and experts in time to study completion and economy of motion. Experts made fewer movements compared to novices and completed the study objectives in less time. In other studies, economy of motion has been well-validated as an assessment tool for procedural expertise, including colonoscopy [25,27-30]. For the colonoscope handle controller, most of the difference in hand motion between experts and novices is attributable to the left hand (Table 2). This result suggests that experts use fewer colonoscope control-head movements and more twisting, pushing, and pulling motions with their right hand. These findings likely reflect experience as practiced endoscopists may use their right hand more, as is consistent with more modern endoscopy educational techniques of using twist and torque over dials [33,34]. This effect may be an advantage with this controller as it may teach learners the fine movements of turning the knobs with one hand, which is not a skill simulated in other common surgical procedures.

There were no differences in the number of button presses or total path length traveled by participants’ hands between the novice and expert groups. Button presses are not a previously validated metric of expertise. We hypothesized that novices may press more buttons unnecessarily, possibly out of desperation or frustration; however, this behavior was not observed. While the difference in path length between groups was not significant, experts did trend toward a smaller path length (Table 2). Perhaps the magnitude of this difference was too small to be detected in the current sample size.

One purported limitation of using video games to assess surgical skills is the potential for video game expertise to bias results [22]. As such, we assessed participants’ past video game experience in the pre-participation survey. In this survey, self-reported game experience was slightly weighted toward novices (Table 1), which if anything would have artificially weakened the magnitude of our findings. To control for video game expertise, each participant played through the game as fast as possible using conventional gaming controls. There was no difference between groups using conventional controls, which provides further evidence that the difference between groups while using the colonoscope is more related to endoscopy expertise rather than to video game experience. We also assessed the correlation between self-reported data and HMA outcomes (Figure 2). Only performance using the mouse and keyboard was associated with past video game and FPS experience. There was no association between performance using the colonoscope controller and past video game experience.

Despite the importance of our findings, there are a few notable limitations. This study provides initial validity evidence for this controller using a single FPS video game. Experts outperform novices using this combination only. We have not assessed whether these skills are generalizable to other games while using this controller, nor have we assessed whether this controller can be used to train basic endoscopy skills. In its current form, this device is compatible with many computer games that require the use of a few buttons, a mouse, and forward/backward controls. Trainees can tailor this adaptable system to their favorite games. However, at present, it is unclear if using a different game would yield training benefits, especially using a different game genre. Based on constructs used in endoscopy, we hypothesize that a flight simulator may be another potential genre to trial.

Other sources of validity evidence recommended for assessing surgical simulation tools have not yet been measured for this novel controller. For this study, licensure and the number of past endoscopies performed were used as a surrogate marker for endoscopy expertise. A scoring system to assess these movements was not used, as existing metrics such as the Global Assessment of Gastrointestinal Endoscopic Skills for Colonoscopy [35] or FES [36] do not apply to this model without separately testing all participants in vivo or using a separate procedural endoscopy simulator. While threshold numbers are regarded as a poor reflection of individual competence [32], they have been used for years as a benchmark and so were measured for our study. Interestingly, the number of self-reported endoscopy procedures performed was correlated with time and number of hand motions using the colonoscope controller (Figure 2C, 2D), supporting the notion that the number of past endoscopies was a good metric with which to determine expertise for this study. The next steps will be to use this machine to train a novice cohort of endoscopists and to correlate performance against some of these other validated metrics in vivo.

Another important limitation is cost. The current prototype used a colonoscope donated by the manufacturer. A new colonoscope is costly. Those who wish to build their own version of this model could save money by purchasing used or refurbished scopes, which are available online starting at $150. As we did not use any colonoscope parts aside from the dials and the tube, a nonfunctioning colonoscope without fiberoptics and lights could be a less expensive viable option. Additional costs include the Leap Motion controller, which can be purchased new for $100. The wood, Styrofoam, tape, and copper pipe used to make the stand were inexpensive. The GameWave computer software is free, and most trainees would have access to a laptop.

In its current form, this model is inexpensive enough that trainees could build their own and is comparable in price to many commercial video gaming systems available. However, the real strength of this apparatus is its potential as an educational tool through which endoscopy trainees can learn new skills while playing. It could be used outside work hours during leisure time and offers an opportunity to do so more conveniently at home rather than in an institutional simulation lab.

## Conclusions

Experts outperformed novices using a modified colonoscope to control an FPS video game with respect to economy of movement and completion time. This result demonstrates that this endoscope-controlled video game shares similar constructs with real-life endoscopy and serves as a first step toward creating an enjoyable alternative to commercially available endoscopy simulators. Future studies should aim to establish repeatability of measurements and correlate training improvements on this model with corresponding improvements in objective endoscopy performance.

## References

[1] Christmas AB, Brintzenhoff RA, Sing RF, Schmelzer TM, Bolton SD, Miles WS, Thomason MH (2009). Resident work hour restrictions impact chief resident operative experience. Am Surg.

[2] Morrissey S, Dumire R, Bost J, Gregory JS (2011). Feasibility of and barriers to continuity of care in US general surgery residencies with an 80-hour duty week. Am J Surg.

[3] Watson DR, Flesher TD, Ruiz O, Chung JS (2010). Impact of the 80-hour workweek on surgical case exposure within a general surgery residency program. J Surg Educ.

[4] Macgregor JM, Sticca R (2010). General surgery residents' views on work hours regulations. J Surg Educ.

[5] Nataraja RM, Webb N, Lopez PJ (2018). Simulation in paediatric urology and surgery. Part 1: an overview of educational theory. J Pediatr Urol.

[6] Bittner JG 4th, Coverdill JE, Imam T, Deladisma AM, Edwards MA, Mellinger JD (2008). Do increased training requirements in gastrointestinal endoscopy and advanced laparoscopy necessitate a paradigm shift? A survey of program directors in surgery. J Surg Educ.

[7] Zendejas B, Ruparel RK, Cook DA (2016). Validity evidence for the Fundamentals of Laparoscopic Surgery (FLS) program as an assessment tool: a systematic review. Surg Endosc.

[8] Mizota T, Anton NE, Huffman EM, Guzman MJ, Lane F, Choi JN, Stefanidis D (2020). Development of a fundamentals of endoscopic surgery proficiency-based skills curriculum for general surgery residents. Surg Endosc.

[9] Desilets DJ, Banerjee S, Barth BA (2011). Endoscopic simulators. Gastrointest Endosc.

[10] Ritter KA, Leifer D, Orabi D, Prabhu A, French J, Lipman JM (2019). How we do it: creation of a low-cost endoscopic skills model for fundamentals of endoscopic surgery training. J Surg Educ.

[11] Walsh CM, Sherlock ME, Ling SC, Carnahan H (2012). Virtual reality simulation training for health professions trainees in gastrointestinal endoscopy. Cochrane Database Syst Rev.

[12] Park J, MacRae H, Musselman LJ, Rossos P, Hamstra SJ, Wolman S, Reznick RK (2007). Randomized controlled trial of virtual reality simulator training: transfer to live patients. Am J Surg.

[13] van Empel PJ, Verdam MG, Strypet M (2012). Voluntary autonomous simulator based training in minimally invasive surgery, residents' compliance and reflection. J Surg Educ.

[14] Schlickum MK, Hedman L, Enochsson L, Kjellin A, Felländer-Tsai L (2009). Systematic video game training in surgical novices improves performance in virtual reality endoscopic surgical simulators: a prospective randomized study. World J Surg.

[15] (2021). Entertainment software association of canada: Real Canadian Gamer Essential Facts 2020. https://theesa.ca/resources/essential-facts/.

[16] Boot WR, Kramer AF, Simons DJ, Fabiani M, Gratton G (2008). The effects of video game playing on attention, memory, and executive control. Acta Psychol (Amst).

[17] Green CS, Bavelier D (2003). Action video game modifies visual selective attention. Nature.

[18] Glassman D, Yiasemidou M, Ishii H, Somani BK, Ahmed K, Biyani CS (2016). Effect of playing video games on laparoscopic skills performance: a systematic review. J Endourol.

[19] Maan ZN, Maan IN, Darzi AW, Aggarwal R (2012). Systematic review of predictors of surgical performance. Br J Surg.

[20] Schlickum MK, Hedman L, Enochsson L, Kjellin A, Felländer-Tsai L (2008). Transfer of systematic computer game training in surgical novices on performance in virtual reality image guided surgical simulators. Studies in Health Technology and Informatics.

[21] Enochsson L, Isaksson B, Tour R, Kjellin A, Hedman L, Wredmark T, Tsai-Felländer L (2004). Visuospatial skills and computer game experience influence the performance of virtual endoscopy. J Gastrointest Surg.

[22] Jalink MB, Goris J, Heineman E, Pierie JP, ten Cate Hoedemaker HO (2014). Construct and concurrent validity of a Nintendo Wii video game made for training basic laparoscopic skills. Surg Endosc.

[23] American Educational Research Association (2014). The Standards for Educational and Psychological Testing. https://www.apa.org/science/programs/testing/standards.

[24] Borgersen NJ, Naur TM, Sørensen SM, Bjerrum F, Konge L, Subhi Y, Thomsen AS (2018). Gathering validity evidence for surgical simulation: a systematic review. Ann Surg.

[25] Ziesmann MT, Park J, Unger B (2015). Validation of hand motion analysis as an objective assessment tool for the Focused Assessment with Sonography for Trauma examination. J Trauma Acute Care Surg.

[26] Grober ED, Roberts M, Shin EJ, Mahdi M, Bacal V (2010). Intraoperative assessment of technical skills on live patients using economy of hand motion: establishing learning curves of surgical competence. Am J Surg.

[27] Ratuapli SK, Ruff KC, Ramirez FC, Wu Q, Mohankumar D, Santello M, Fleischer DE (2015). Kinematic analysis of wrist motion during simulated colonoscopy in first-year gastroenterology fellows. Endosc Int Open.

[28] Holden MS, Wang CN, MacNeil K, Church B, Hookey L, Fichtinger G, Ungi T (2018). Objective assessment of colonoscope manipulation skills in colonoscopy training. Int J Comput Assist Radiol Surg.

[29] Uemura M, Tomikawa M, Kumashiro R (2014). Analysis of hand motion differentiates expert and novice surgeons. J Surg Res.

[30] Mason JD, Ansell J, Warren N, Torkington J (2013). Is motion analysis a valid tool for assessing laparoscopic skill?. Surg Endosc.

[31] Datta V, Mackay S, Mandalia M, Darzi A (2001). The use of electromagnetic motion tracking analysis to objectively measure open surgical skill in the laboratory-based model. J Am Coll Surg.

[32] Ekkelenkamp VE, Koch AD, de Man RA, Kuipers EJ (2016). Training and competence assessment in GI endoscopy: a systematic review. Gut.

[33] Lee SH, Park YK, Cho SM, Kang JK, Lee DJ (2015). Technical skills and training of upper gastrointestinal endoscopy for new beginners. World J Gastroenterol.

[34] Lee SH, Park YK, Lee DJ, Kim KM (2014). Colonoscopy procedural skills and training for new beginners. World J Gastroenterol.

[35] Vassiliou MC, Kaneva PA, Poulose BK (2010). Global Assessment of Gastrointestinal Endoscopic Skills (GAGES): a valid measurement tool for technical skills in flexible endoscopy. Surg Endosc.

[36] Vassiliou MC, Dunkin BJ, Fried GM (2014). Fundamentals of endoscopic surgery: creation and validation of the hands-on test. Surg Endosc.

